# Supplementary material for: What the geological past can tell us about the future of the ocean’s twilight zone
Source: Nat Commun. 2023 Apr 27;14:2376. doi: 10.1038/s41467-023-37781-6 (PMC10140295; doi:10.1038/s41467-023-37781-6)
Supplement: Supplementary file 1 — supplementary information [file 41467_2023_37781_MOESM1_ESM.pdf]

Supplementary Information for

# What the geological past can tell us about the future of the ocean's twilight zone

AUTHORS

Katherine A. Crichton[1]*†, Jamie D. Wilson[2,3], Andy Ridgwell[4], Flavia Boscolo-Galazzo[1]‡, Eleanor H. John[1], Bridget S. Wade[5], Paul. N. Pearson[1]

AFFILIATIONS

[1] School of Earth and Environmental Science, Cardiff University, Cardiff, UK.
[2] School of Earth Sciences, University of Bristol, Bristol, UK.
[3] Department of Earth, Ocean and Ecological Sciences, University of Liverpool, Liverpool UK.
[4] Department of Earth and Planetary Sciences, University of California, Riverside, CA, USA.
[5] Department of Earth Sciences, University College London, London, UK.
† Now at Department of Geography, University of Exeter, Exeter, UK.
‡ Now at MARUM, University of Bremen, Germany.

*Corresponding author: k.a.crichton@exeter.ac.uk

**This PDF file includes:**

Supplementary figures. S1 to S8

A. Supplementary discussion on $\delta^{13}C$ model-data comparison
B. Ocean circulation in cGENIE simulations
C. Future simulations comparison to CMIP models
D. Including temperature dependent remineralisation of DOM

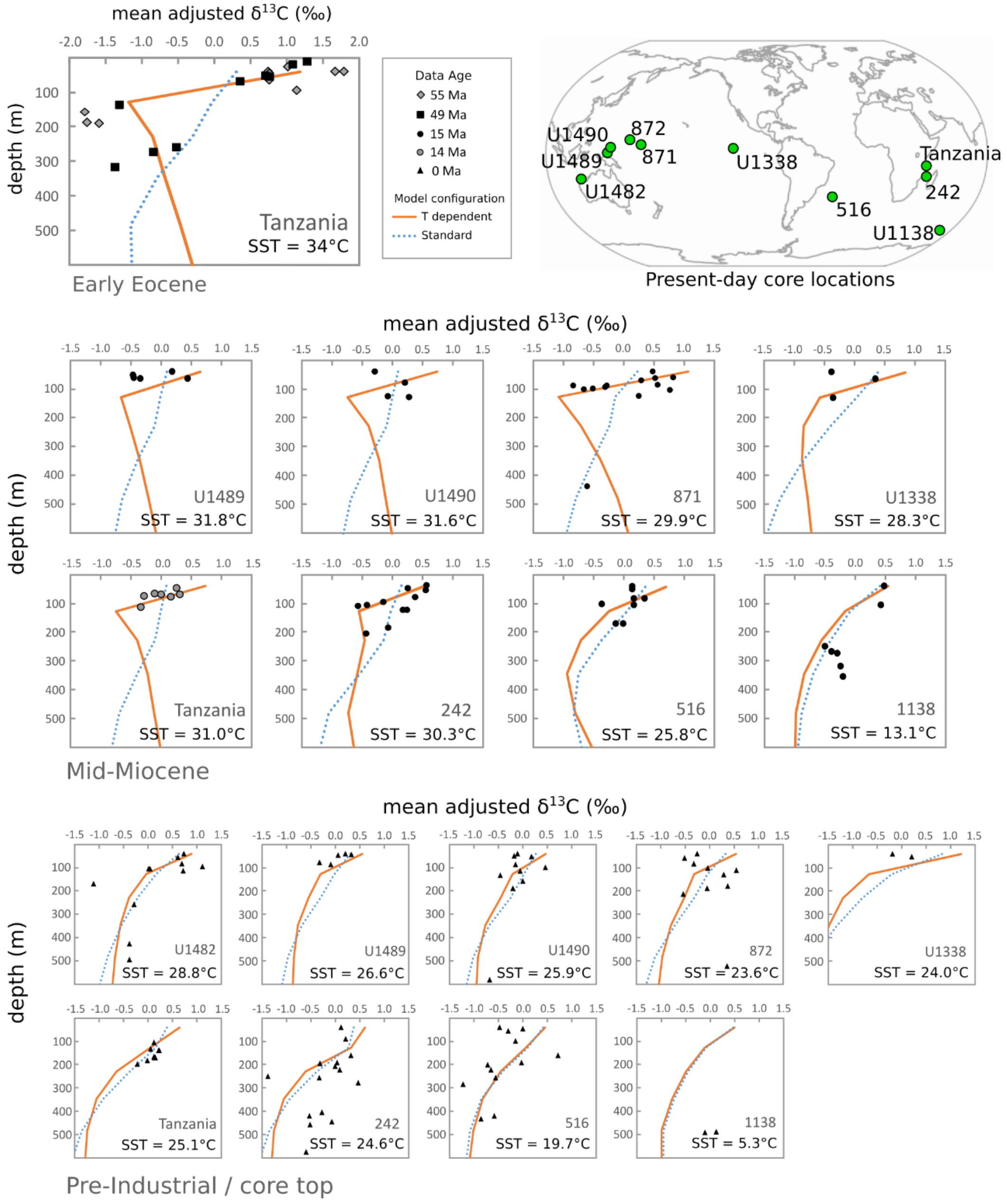


***Fig S1, Comparison of δ$^{13}$C:depth profiles taken from the cGenie model vs. individual planktonic foraminifera data*** *used in the main text. Data is for planktonic foraminifera in the size range 250 to 355 um only, and data and model values are corrected to the mid of their range of δ$^{13}$C from 0m to 228m depth. Model profiles are extracted for model grid points corresponding to the paleo-locations of the data, and modelled sea surface temperature for that location is labelled on each. Data for the early Eocene is from (9), data for the mid-Miocene and Pre-Industrial is from (2). Full model-data discussion for the early Eocene is available in (10), and for the mid-Miocene to Pre-Industrial in (2), including the methods used to derive data depths.*

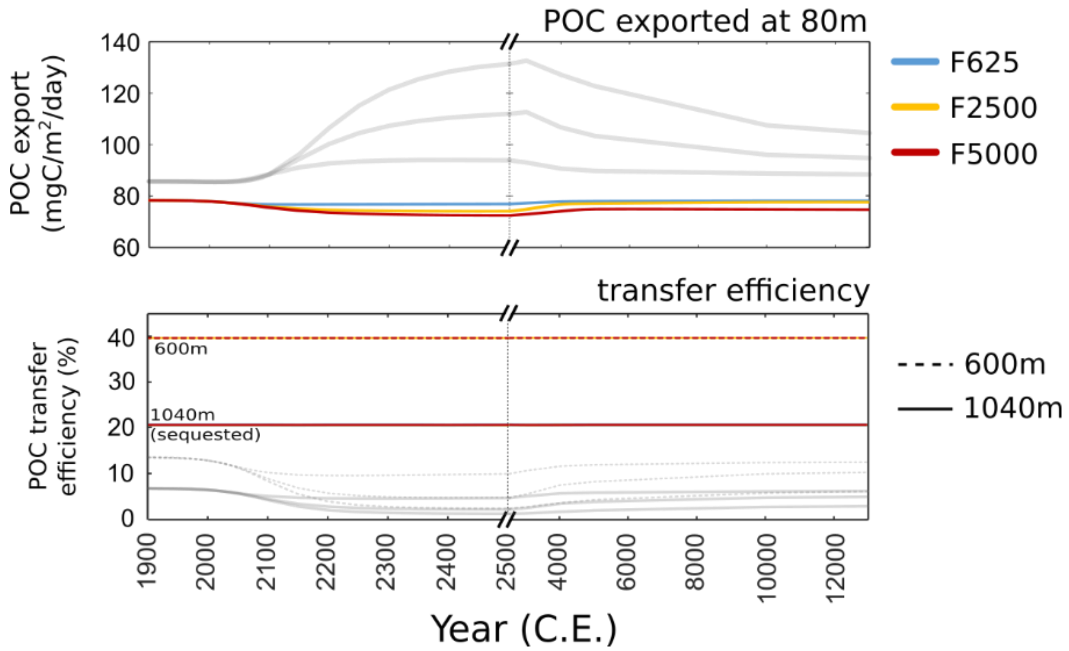


***Fig S2, Projected future scenarios using the standard model configuration*** *(without Biological Carbon Pump temperature dependency) for mean Particulate Organic Carbon (POC) export and mean transfer efficiency for low-latitudes. The temperature dependent model outputs discussed in the main text are shown as light grey lines for comparison.*

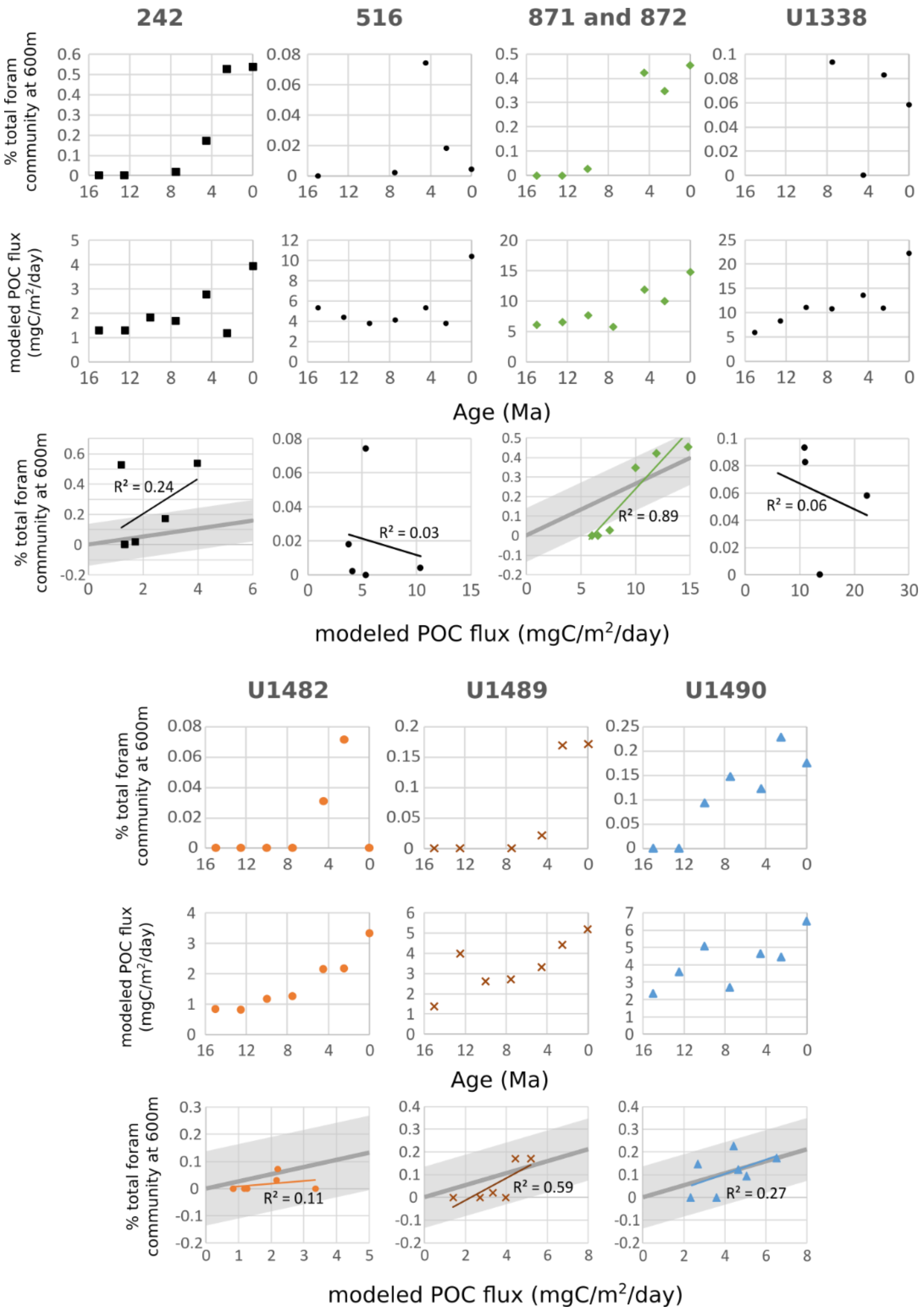


***Fig S3, Data used to create the POC-abundance model***. *Site-specific abundance (plotted as % of community) at 600m depth, and site-specific modelled Particulate Organic Carbon (POC) flux data from (2). Grey line is the general low-latitude POC-abundance model (main text Fig. 4, with the grey shaded region the one sigma error (one standard deviation)), colored lines are linear regression per site with* $R^2$ *marked on each. Only data where* $R^2$ *is greater than 0.1 is included to create the general low-latitude POC-abundance model.*

## A. **Supplementary discussion on δ$^{13}$C model-data comparison**

*Impact of Ocean Circulation*

The effect of ocean circulation changes on δ$^{13}$C in the absence of a metabolic temperature response is illustrated by the 'standard' model. In this configuration, δ$^{13}$C profiles are driven by both changes in physical ocean circulation and also by the different distribution of surface nutrients. Surface nutrients exert a control on export production (along with other parameters, see *53*), which in turn determines the surface δ$^{13}$C of DIC due to the effect of fractionation during photosynthesis (along with air-sea gas exchange rates, the atmospheric ratio of carbon-12 to carbon-13, and the remineralisation of dissolved organic matter in the surface ocean). In the water column, the rate of remineralisation is fixed and dictated according to a global remineralisation vs depth curve (*1*). The effect of ocean circulation on δ$^{13}$C changes starts to become more important as the POC flux reduces with depth; the relative contribution of ocean circulation becomes more important deeper in the water column.

In contrast in the temperature-dependent model, remineralisation rates are no longer globally uniform. Now, remineralisation rates are dependent on local water temperature. Thus, per unit export of POC, more POC is remineralised nearer to the surface in warmer waters than in colder waters. This exerts a control on δ$^{13}$C, with a steeper near-surface δ$^{13}$C-depth gradient in the low latitude warm waters in the temperature-dependent model that is not evident in the standard model (Fig. S4), which is especially true in the warmest paleo condition, the early Eocene.

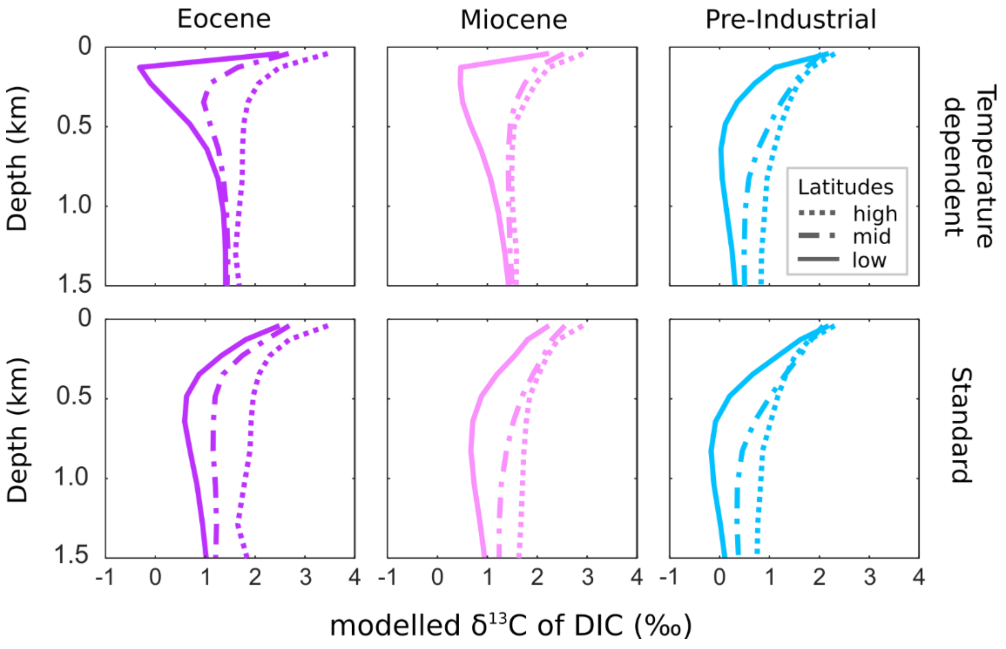


***Fig S4, δ$^{13}$C-depth profiles of the cGENIE model for mean high, mid and low-latitudes*** *for the temperature-dependent cGENIE model (top) and the standard cGENIE model (bottom). The temperature-dependence of metabolic rates in the biological pump have a strong impact on ocean δ$^{13}$C-depth profiles, especially in near-surface waters. DIC is Dissolve Inorganic Carbon.*

*Model-data comparison at different sites*

In mid-latitude waters, such as Site 516 (see Fig. S1), the difference between the $\delta^{13}C$-depth curves of the temperature-dependent (Tdep) to the standard model is far less evident than for the warmer low-latitude waters. The remineralisation curves of the temperature-dependent model are partly controlled by local water temperature, so mid-latitude waters with temperatures that are similar to the global mean may see remineralisation curves that are also similar to the global mean. The global mean $\delta^{13}C$-depth curves for the Tdep and the standard model are not identical as they are tuned differently (*1*). For the Modern simulation, the differences between the Tdep and standard model $\delta^{13}C$-depth curves are at their minimum (although the low-latitude sites – all but sites 516 and 1138 – still do clearly show sharper near-surface gradients for Tdep). The uncertainty in the foraminifera depth and $\delta^{13}C$ data in the present (Modern) is such that the patterns underlying metabolic-temperature dependence, and its effect on $\delta^{13}C$ changes with depth, only become clearly evident by considering the paleo model and data conditions.

It must be noted that our datapoints represent open-ocean conditions at their paleo locations, but that each point should not be considered representative of its ocean-basin, due to differences in water temperatures, which control the remineralisation rates. Site 516 is a mid-latitude site, that although located in the South Atlantic would not be representative of conditions in (for example) the low-latitude South Atlantic which is warmer.

## B. Ocean circulation in cGENIE simulations

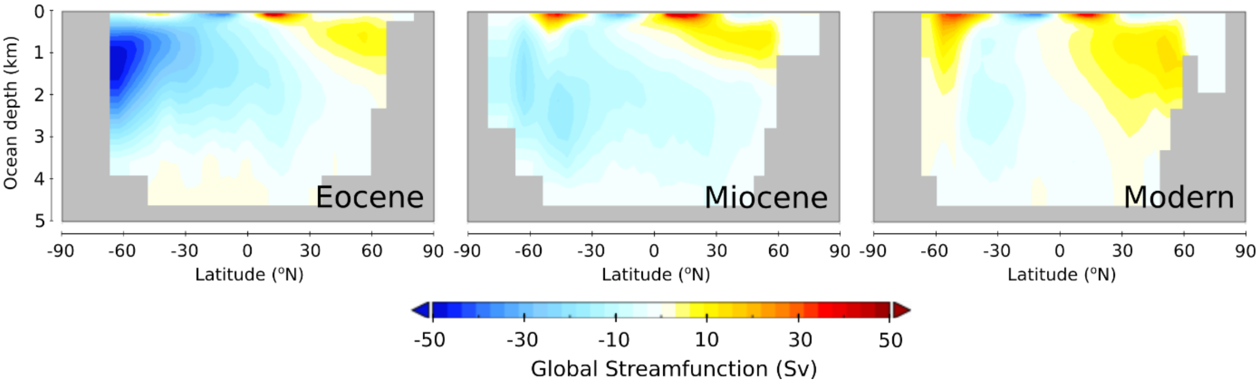


***Fig S5. Global streamfunctions for the early Eocene, mid-Miocene and Modern (Present) model configurations****. Global ocean circulation patterns are a function of ocean bathymetry, continental configuration, global wind fields, and climate forcing.*

Ocean circulation states in the equilibrium cGENIE model simulations depend on climate forcing, including $pCO_2$ and windfields, together with continental configuration. In the paleo-simulations for the early Eocene and the mid-Miocene, the global streamfunction shows reduced Northern deep-water formation compared to the Modern, which has a vigorous Atlantic Meridional Overturning Circulation. Instead, the Eocene exhibits a vigorous overturning from the Southern Ocean (that is ice-free), a feature that becomes muted by the (Fig. S5). These circulation states exert a control on the shape of the thermocline in ocean waters (Fig. 2 main text), and on the distributions of dissolved matter in cGENIE. As such, they also influence the $\delta^{13}C$-depth curves (as discussed above).

For the mid-Miocene model-data comparison carried out in (*2*), the simulated temperature-depth gradient was used to determine the depth-habitat of the foraminifera data. Using present-day data, we derived a relationship between variation in $\delta^{18}O_{sw}$ and salinity, and used this relationship to determine the paleo-ocean water $\Delta\delta^{18}O_{sw}$ field from modelled salinity. We combined this $\Delta\delta^{18}O_{sw}$ field with modelled ocean temperature to solve the calcite paleotemperature equation (*8*), and produced $\Delta\delta^{18}O_{cc}$-depth curves for the data sites, pinning the surface $\delta^{18}O_{cc}$ to the shallowest (lowest $\delta^{18}O_{cc}$) foraminifera, allowing us to match the rest of the measured species-specific $\delta^{18}O_{cc}$ to this curve and read off their depth-habitat. What then are the implications for uncertainty in the modelled paleo thermocline? If the actual paleo thermocline was steeper than simulated (suggested as a possibility for the early-Eocene in *3*), then the true habitat depths of the foraminifers would be shallower than those shown in Fig. S1 In turn, this would mean even steeper re-constructed near-surface $\delta^{13}C$-depth gradients, and a stronger metabolic temperature-dependence of the biological pump than we have assumed. Conversely, if the actual paleo base of the thermocline was deeper than simulated (i.e. and a less-steep thermocline), the foraminifers would in reality be living deeper in the water column, and the actual near-surface $\delta^{13}C$-depth gradients would be less-steep than assumed.

Could the true foraminifera data in reality be closer to the standard model $\delta^{13}C$-depth curves for the Miocene in low-latitudes? For this to happen, the base of the thermocline would need to be so

deep that at depths of 500 m temperatures were still ~25°C, which seems highly unlikely given deep ocean temperatures of 8-11°C (in our modelled low-latitude mid-Miocene, waters at 600m are around 12°C (Fig. 2 main text)). Indeed, for the modern ocean and away from the poles, cGENIE tends to slightly under-estimate the steepness of the upper thermocline – likely due to relatively low vertical resolution and fixed vertical diffusivity with depth (although over the upper 500 m, the mean gradient is approximately correct). Hence, if anything, uncertainties in the modelled paleo thermocline may underestimate the role of temperature-dependent remineralization.

A full analysis of the impacts of significant differences between the modelled past thermocline and the “real” past thermocline would need to be the subject of a separate study, because of the effects on local metabolic rates, dissolved organic matter distribution, global heat distribution, etc.

The transient future forcings have a strong impact on ocean circulation, primarily due to the rate of surface heating and hence density gradients, as well as impacts on salinity through changes in evaporation. The net effect is that the AMOC, which dominates present-day circulation, reduces in strength with time (Fig S6). This effect is strongest under the higher $CO_2$ forcing, but all three projections drive weakening and shoaling of the AMOC the model year 2100 (illustrated by the Atlantic streamfunction in Fig. S6). By 2200, for the mid and high forcings, the AMOC has effectively completely collapsed. This timescale is similar to that shown in more complex models (4), where an AMOC collapse is seen around 300 years after an abrupt doubling of $CO_2$ (since 1990 levels) occurs, and where model biases for a stable AMOC are corrected.

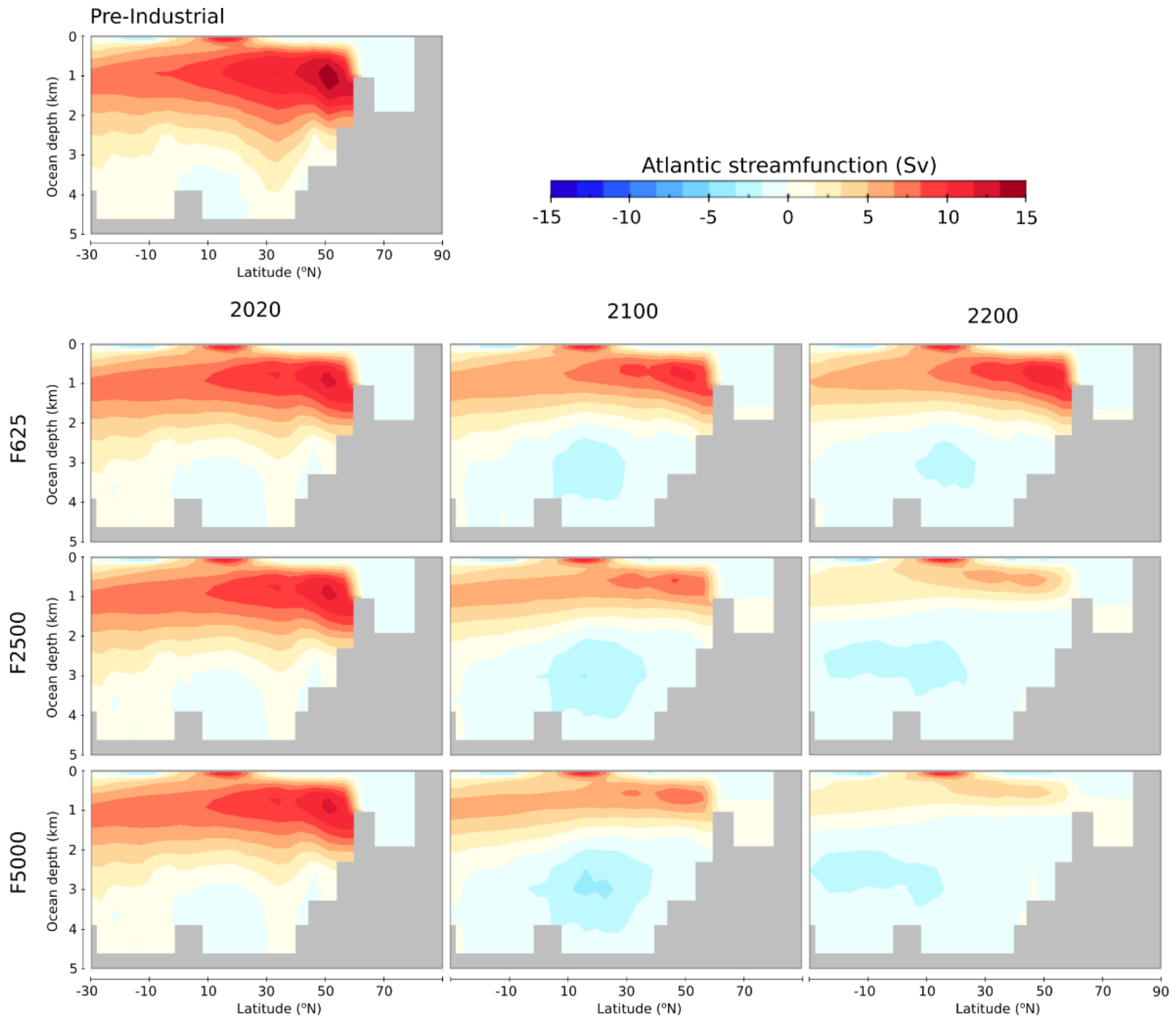


*Fig S6. The basin-averaged Atlantic overturning circulation in cGENIE future projections.*

## C. Future simulations: comparison of cGENIE and CMIP models

In light of the low spatial resolution and lack of inter-annual variability in the model (Methods), the future scenarios modelled in cGENIE should be looked at as illustrative, and intended for making a first-order comparison between past (and present) and future. Despite this, the simulated gross ocean uptake of anthropogenic $CO_2$ from the atmosphere in cGENIE is within observational uncertainty (as shown in 5). The projected responses in large-scale physical ocean circulation to anthropogenic $CO_2$ forcing in cGENIE also show similar characteristics to the multi-model mean response of more complex CMIP5 models, as do projected changes in sub-surface oxygen concentration (summarized below). To maintain consistency with published paleo model-data analyses, we adopted a simplified single-nutrient control on biological export and planktonic ecosystem dynamics and which also differs from the model complex ecosystem dynamics considered in the CMIP5 models. Finally, we do not account for changes in ocean volume or salinity that may arise from changes in global land-ice volume (the forcings we apply are high enough to destabilize (F625 low forcing) or melt significant amounts of the Antarctic ice sheet on the timescales we consider (F2500 mid and F5000 high forcing) (6)). Our models simulations are not therefore intended to constitute robust 'predictions' per se, but rather should be considered as a first attempt to explore how severe such impacts might be under a broad range of future scenarios.

*Ocean Circulation*

As an evaluation of the physical ocean (and specifically overturning circulation in the North Atlantic) response to transient $CO_2$ forcing, we compare the cGENIE F5000 output to the model-mean of CMIP5 models under the RCP 8.5 scenario. The F5000 $CO_2$ forcing is not exactly the same $CO_2$ concentration pathway as RCP 8.5, but we assume them here to be comparable high-maximum and fast-rising $CO_2$ forcing scenarios. The $CO_2$ concentrations for the years 2000 to 2100 are shown in Fig. S7, along with the maximum overturning circulation in the North Atlantic for the CMIP5 multi-model mean (7), and for the cGENIE tdep F5000 forcing in this study. Also shown are the depth structure of the streamfunction at 40°N (latitudinal basin-mean).

Maximum overturning is lower in cGENIE than in the CMIP5 models, attributable to the large grid cell size in cGENIE. In response to $CO_2$ forcing, cGENIEs overturning is also slightly more sensitive than the CMIP5 multi-model mean. Similar patterns occur between cGENIE and the CMIP5 models in terms of the time-dependent depth structure of the streamfunction (at 40°N) with a shoaling of the AMOC and reduction in strength of similar magnitudes over this 100-year period.

*Subsurface Oxygen Concentrations*

Changes in subsurface oxygen will integrate changes in ocean circulation (and stratification), export production, and remineralization, and as such represents a strong test of similarities in model response. We find that changes in subsurface oxygen show similar patterns to the CMIP6 multi-model mean for the cGENIE standard model simulation (Fig. S8). The North Pacific reveals strongest $O_2$ depletion under warming, the northern Indian Ocean shows increases in oxygen concentration. The CMIP6 models have various treatments for temperature dependence, with some models accounting in some way for temperature effects on remineralisation rates and others not (8). When including temperature dependence on POM "Tdep (POM)" (and on both

POM and DOM remineralisation rates “Tdep (DOM POM)”) sub-surface oxygen depletion occurs in almost all locations, including low latitude locations that in the standard model show an increase in oxygen concentration (for example the northern Indian Ocean, the low-latitude Atlantic, Fig. S8). It is noteworthy that the areas where CMIP6 models show disagreement (areas without black dots in Fig. S5a) are the areas where temperature dependence results in oxygen depletion (rather than oxygen increase in the standard model), these areas are the Indian Ocean, the west Pacific low latitudes, and the low latitude tropical Atlantic. This could account for some of the CMIP6 model disagreement, if some models include temperature dependent remineralisation and some do not. cGENIE exhibits less depletion of sub-surface oxygen in the North Atlantic ocean compared to CMIP6 models’ ensemble. This is likely related to differences in the ventilation of near-surface waters linked to AMOC strength (as discussed above).

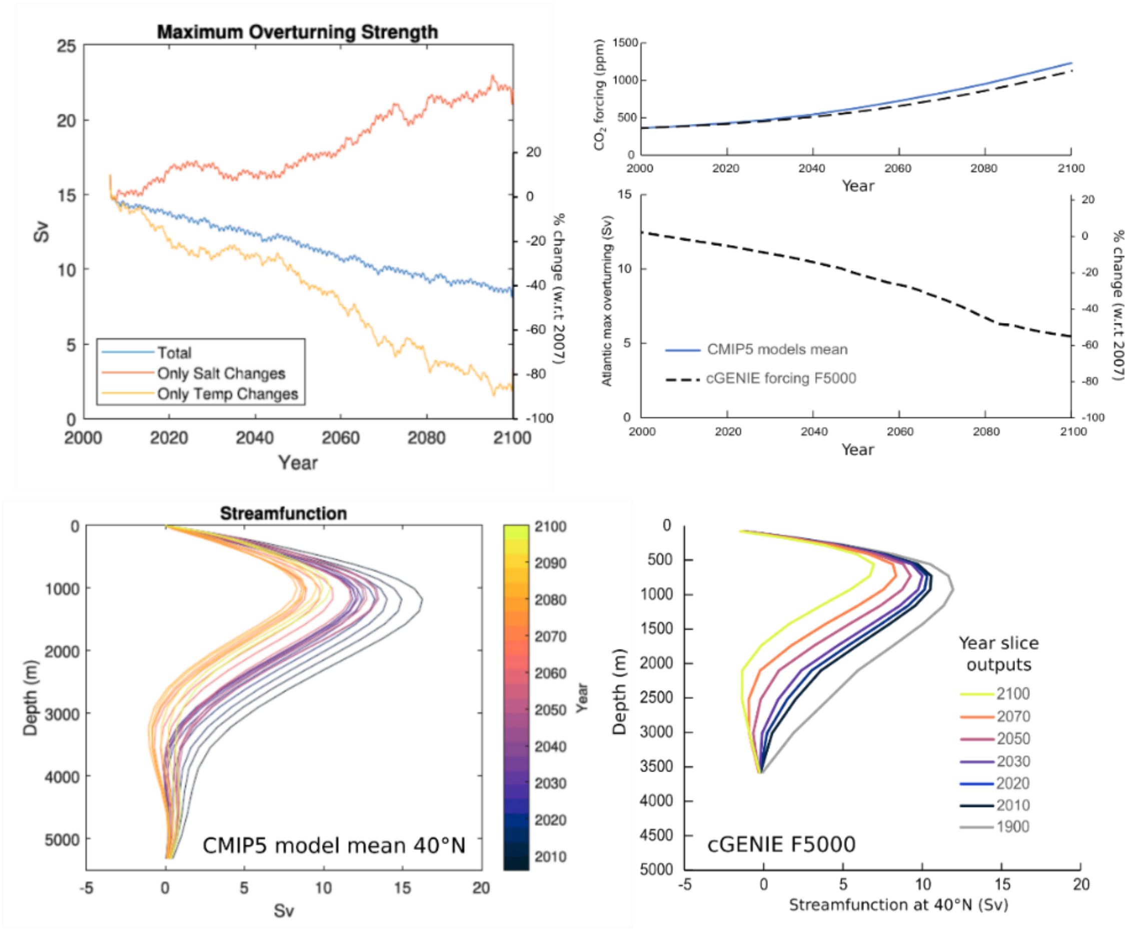


***Fig S7, Characteristics of Atlantic overturning circulation for the CMIP5 RCP 8.5 scenario and the cGENIE F5000*** *simulation in this study. The cGENIE circulation response to $CO_2$ forcing is similar to the multi-model mean of CMIP5 models[7]. Top left, Maximum overturning strength multi-model mean for CMIP5[7]. Top right, $CO_2$ forcing scenarios implemented in CMIP5 models (blue line) and that used in cGENIE high emissions forcing (dashed line), and just below cGENIE maximum overturning strength. Bottom left, mean watercolumn streamfunction for CMIP5 models[7]. Bottom right, cGENIE high emissions scenario mean watercolumn streamfunction. Figures from Levang, S.J. and Schmitt, R.W., What causes*

*the AMOC to weaken in CMIP5?. J. Clim, 33(4), pp.1535-1545. DOI: https://doi.org/10.1175/JCLI-D-19-0547.1 (2020)*[7] *© American Meteorological Society. Used with permission.*

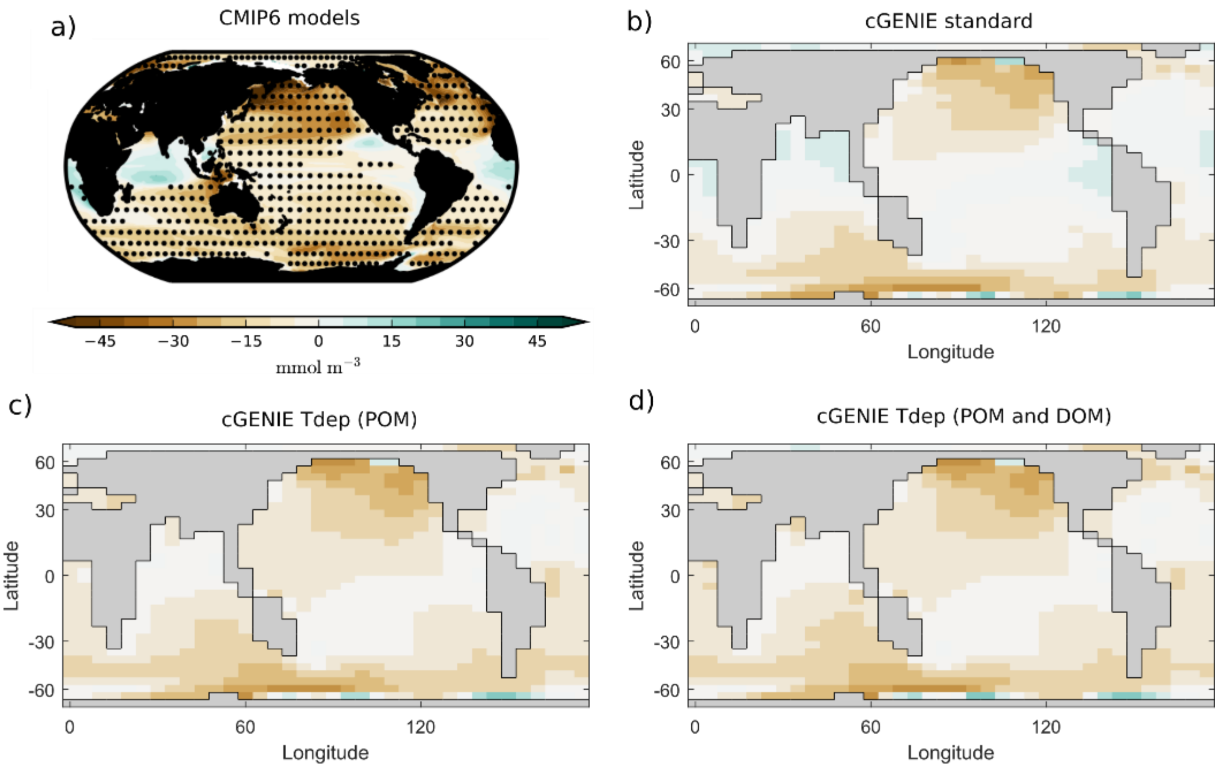


***Fig S8, Change in subsurface oxygen saturation, comparison of CMIP6 models under SSP5-8.5 to the cGENIE F5000 simulation*** *for the years 2080-2099 anomaly with respect to the 1995-2014 baseline (for CMIP6). a) CMIP6 multi-model mean, with areas of model-agreement represented with black dots (reproduced from Kwiatkowski, L., et al., Twenty-first century ocean warming, acidification, deoxygenation, and upper-ocean nutrient and primary production decline from CMIP6 model projections. Biogeosciences,* ***17****(13), pp.3439-3470. (2020)). Panels b), c), and d) are cGENIE model output with different Biological Carbon Pump (BCP) temperature dependence setting. b) cGENIE BCP without temperature dependence; c) Tdep (POM) is the cGENIE temperature dependent Particulate Organic Matter biological carbon pump model; d) Tdep (POM and DOM) is the cGENIE temperature dependent Particulate Organic Matter and Dissolved Organic Matter biological carbon pump model.*

## D. Including temperature dependent remineralisation of Dissolved Organic Matter

The studies which we cite in this work, and those we used to make the POC-abundance model, account for temperature dependence in the rate of degradation of POM, but not of DOM. In Crichton et al. (*1*), a temperature-dependence DOM scheme was described for cGENIE, so we also explore in this study whether there is any substantive impact on future projections of temperature dependence of DOM.

Including Tdep DOM slightly ameliorates the effect on oxygen of warming in F5000, with a maximum improvement of just under 11% in the dissolved oxygen minimum compared to the Tdep (POM, as in main text) in year 2250, and a deeper oxygen minimum depth of almost 50m in year 2080. All other differences are smaller than these. The effect on POC export is a mean difference of -2.5% when Tdep DOM is included. There is also a small effect on POC flux at 600m, with around 2.5% less POC delivered compared to the Tdep POM simulation (that described in the main text) for the F5000 simulation.

These differences are similar in scale to those identified in (1) for the instrumental period, where although including temperature dependent DOM does have a small impact, it is dwarfed by the impact that temperature dependent POM has on the action of the biological pump under warming scenarios.

**References**


1. Crichton, K.A., Wilson, J.D., Ridgwell, A. and Pearson, P.N., Calibration of temperature-dependent ocean microbial processes in the cGENIE. muffin (v0. 9.13) Earth system model. *Geosci. Mod. Dev.*, 14(1), 125-149. DOI: https://doi.org/10.5194/gmd-14-125-2021, (2021).
2. Boscolo-Galazzo, F., Crichton, K.A., Ridgwell, A., Mawbey, E.M., Wade, B.S. and Pearson, P.N., 2021. Temperature controls carbon cycling and biological evolution in the ocean twilight zone, *Science*, **371**(6534), 1148-1152, DOI: https://doi.org/10.1126/science.abb6643, (2021).
3. John, E.H., Wilson, J.D., Pearson, P.N., Ridgwell, A. Temperature-dependent remineralization and carbon cycling in the warm Eocene oceans. *Palaeogeog, Palaeoclim., Palaeoecol.* **413**, 158-166, https://doi.org/10.1016/j.palaeo.2014.05.019, (2014).
4. Liu, W., Xie, S.P., Liu, Z. and Zhu, J., Overlooked possibility of a collapsed Atlantic Meridional Overturning Circulation in warming climate. *Sci. Adv.*, **3**(1), p.e1601666. DOI: https://doi.org/10.1126/sciadv.16016 (2017).
5. Cao, L., Eby, M., Ridgwell, A., Caldeira, K., Archer, D., Ishida, A., Joos, F., Matsumoto, K., Mikolajewicz, U., Mouchet, A. and Orr, J.C., The role of ocean transport in the uptake of anthropogenic CO2. *Biogeosciences*, **6**(3), 375-390, DOI: https://doi.org/10.5194/bg-6-375-2009, (2009).
6. Winkelmann, R., Levermann, A., Ridgwell, A. and Caldeira, K., Combustion of available fossil fuel resources sufficient to eliminate the Antarctic Ice Sheet, *Sci. Advances*, **1**(8), p.e1500589. DOI: https://doi.org/10.1126/sciadv.1500589, (2015).
7. Levang, S.J. and Schmitt, R.W., What causes the AMOC to weaken in CMIP5?. *J. Clim*, **33**(4), pp.1535-1545. DOI: https://doi.org/10.1175/JCLI-D-19-0547.1 (2020).
8. Séférian, R., Berthet, S., Yool, A., Palmieri, J., Bopp, L., Tagliabue, A., Kwiatkowski, L., Aumont, O., Christian, J., Dunne, J. and Gehlen, M., Tracking improvement in simulated marine biogeochemistry between CMIP5 and CMIP6. *Curr. Clim. Change Rep.*, **6**(3), pp.95-119. DOI: https://doi.org/10.1007/s40641-020-00160-0, (2020).
9. Pearson, P.N., van Dongen, B.E., Nicholas, C.J., Pancost, R.D., Schouten, S., Singano, J.M., Wade, B.S., Stable warm tropical climate through the Eocene epoch, *Geology*, **35**, 211–214, DOI: https://doi.org/10.1130/G23175A.1, (2007).
10. John, E.H., Wilson, J.D., Pearson, P.N., Ridgwell, A. Temperature-dependent remineralization and carbon cycling in the warm Eocene oceans. *Palaeogeog, Palaeoclim., Palaeoecol.* **413**, 158-166, https://doi.org/10.1016/j.palaeo.2014.05.019, (2014).
11. Kwiatkowski, L., Torres, O., Bopp, L., Aumont, O., Chamberlain, M., Christian, J.R., Dunne, J.P., Gehlen, M., Ilyina, T., John, J.G. and Lenton, A., Twenty-first century ocean warming, acidification, deoxygenation, and upper-ocean nutrient and primary production decline from CMIP6 model projections. *Biogeosciences*, **17**(13), pp.3439-3470. DOI: https://doi.org/10.5194/bg-17-3439-2020, (2020).
