# Supplementary material for: What the geological past can tell us about the future of the ocean’s twilight zone
Source: Nat Commun. 2023 Apr 27;14:2376. doi: 10.1038/s41467-023-37781-6 (PMC10140295; doi:10.1038/s41467-023-37781-6)
Supplement: Supplementary file 3 — Reporting Summary [file 41467_2023_37781_MOESM3_ESM.pdf]

nature portfolio

Corresponding author(s): Dr. Katherine A Crichton

Last updated by author(s): Mar 17, 2023

Reporting Summary

Nature Portfolio wishes to improve the reproducibility of the work that we publish. This form provides structure for consistency and transparency in reporting. For further information on Nature Portfolio policies, see our Editorial Policies and the Editorial Policy Checklist.

Statistics

For all statistical analyses, confirm that the following items are present in the figure legend, table legend, main text, or Methods section.

n/a	Confirmed	
x		The exact sample size (n) for each experimental group/condition, given as a discrete number and unit of measurement
x		A statement on whether measurements were taken from distinct samples or whether the same sample was measured repeatedly
x		The statistical test(s) used AND whether they are one- or two-sided<br>*Only common tests should be described solely by name; describe more complex techniques in the Methods section.*
x		A description of all covariates tested
x		A description of any assumptions or corrections, such as tests of normality and adjustment for multiple comparisons
	x	A full description of the statistical parameters including central tendency (e.g. means) or other basic estimates (e.g. regression coefficient) AND variation (e.g. standard deviation) or associated estimates of uncertainty (e.g. confidence intervals)
x		For null hypothesis testing, the test statistic (e.g. F, t, r) with confidence intervals, effect sizes, degrees of freedom and P value noted<br>*Give P values as exact values whenever suitable.*
x		For Bayesian analysis, information on the choice of priors and Markov chain Monte Carlo settings
x		For hierarchical and complex designs, identification of the appropriate level for tests and full reporting of outcomes
x		Estimates of effect sizes (e.g. Cohen's d, Pearson's r), indicating how they were calculated

Our web collection on statistics for biologists contains articles on many of the points above.

Software and code

Policy information about availability of computer code

Data collection	CODE AVAILABILITY<br>The code for the version of the 'muffin' release of the cGENIE Earth system model used in this paper, is tagged as v0.9.26, and is assigned a DOI: https://doi.org/10.5281/zenodo.5140571. Configuration files for the specific experiments presented in the paper can be found in the directory: genie-userconfigs/MS/crichtonetal.SciAdv.2021. Details of the experiments, plus the command line needed to run each one, are given in the readme.txt file in that directory. All other configuration files and boundary conditions are provided as part of the code release. A manual detailing code installation, basic model configuration, tutorials covering various aspects of model configuration and experimental design, plus results output and processing, is assigned a DOI: https://doi.org/10.5281/zenodo.5130672.
Data analysis	CODE AVAILABILITY<br>The code for the version of the 'muffin' release of the cGENIE Earth system model used in this paper, is tagged as v0.9.26, and is assigned a DOI: https://doi.org/10.5281/zenodo.5140571. Configuration files for the specific experiments presented in the paper can be found in the directory: genie-userconfigs/MS/crichtonetal.SciAdv.2021. Details of the experiments, plus the command line needed to run each one, are given in the readme.txt file in that directory. All other configuration files and boundary conditions are provided as part of the code release. A manual detailing code installation, basic model configuration, tutorials covering various aspects of model configuration and experimental design, plus results output and processing, is assigned a DOI: https://doi.org/10.5281/zenodo.5130672.

For manuscripts utilizing custom algorithms or software that are central to the research but not yet described in published literature, software must be made available to editors and reviewers. We strongly encourage code deposition in a community repository (e.g. GitHub). See the Nature Portfolio guidelines for submitting code & software for further information.

Data

Policy information about availability of data

All manuscripts must include a data availability statement. This statement should provide the following information, where applicable:

- Accession codes, unique identifiers, or web links for publicly available datasets
- A description of any restrictions on data availability
- For clinical datasets or third party data, please ensure that the statement adheres to our policy

DATA AVAILABILITY
All data used is available in the text or supplementary materials.

Human research participants

Policy information about studies involving human research participants and Sex and Gender in Research.

Reporting on sex and gender	not relevant to this study
Population characteristics	not relevant to this study
Recruitment	not relevant to this study
Ethics oversight	not relevant to this study

Note that full information on the approval of the study protocol must also be provided in the manuscript.

Field-specific reporting

Please select the one below that is the best fit for your research. If you are not sure, read the appropriate sections before making your selection.

☐ Life sciences ☐ Behavioural & social sciences ☒ Ecological, evolutionary & environmental sciences

For a reference copy of the document with all sections, see nature.com/documents/nr-reporting-summary-flat.pdf

Ecological, evolutionary & environmental sciences study design

All studies must disclose on these points even when the disclosure is negative.

Study description	In this study, we start by reviewing the fundamental differences in past mesopelagic plankton communities under warmer climatic conditions and show how Earth system modelling of the Biological Carbon Pump (BCP) and associated carbon isotopes has helped explain these patterns. We then build on this, deliberately using the same model applied to the past, to investigate what changes may have already occurred since the pre-industrial period as well as illustrate what may happen in the future. We focus in particular on low-latitude TZ habitats; the high latitude oceans are more strongly affected by vigorous horizontal and vertical mixing processes that can overprint and obscure the temperature-dependent effects under investigation, as well as generally having a much less complete paleo record. We contrast past, present and future ocean conditions and critically discuss to what extent observations of past steady state conditions can be used in inferring impacts in a dynamically changing present and future world. We do not collect new data for this study, but only apply previously published data with an Earth System Models simulations.
Research sample	Novel work in this study is the use of an Earth system model to compare past and future warm climates. Included is a summary of previously studies (published) which involved the sampling and isotopic measurement of planktonic foraminifera from ocean cores. We did not create new datasets for this study.
Sampling strategy	we didnt sample anything new for this study
Data collection	we didnt collect any new data from ocean sediments etc in this study - we applied previously published data to results from an Earth System Model
Timing and spatial scale	no new data of this kind was collected for this study
Data exclusions	we didnt collect new data for this study, so this does not apply
Reproducibility	we didnt collect new data for this study, but the model code and instructions to re-run our model simulations are available in the main text. Citations of work where paleontological data was collected are in the reference list and clearly cited
Randomization	we didnt collect new data for this study

Blinding | we didnt collect new data for this study

Did the study involve field work? ☐ Yes ☒ No

Reporting for specific materials, systems and methods

We require information from authors about some types of materials, experimental systems and methods used in many studies. Here, indicate whether each material, system or method listed is relevant to your study. If you are not sure if a list item applies to your research, read the appropriate section before selecting a response.

Materials & experimental systems

n/a	Involved in the study
☒	☐ Antibodies
☒	☐ Eukaryotic cell lines
☒	☐ Palaeontology and archaeology
☒	☐ Animals and other organisms
☒	☐ Clinical data
☒	☐ Dual use research of concern

Methods

n/a	Involved in the study
☒	☐ ChIP-seq
☒	☐ Flow cytometry
☒	☐ MRI-based neuroimaging
